# Metabolomics and its Applications in Cancer Cachexia

**DOI:** 10.3389/fmolb.2022.789889

**Published:** 2022-02-07

**Authors:** Pengfei Cui, Xiaoyi Li, Caihua Huang, Qinxi Li, Donghai Lin

**Affiliations:** ^1^ College of Food and Pharmacy, Xuchang University, Xuchang, China; ^2^ Xuchang Central Hospital, Xuchang, China; ^3^ Department of Physical Education, Xiamen University of Technology, Xiamen, China; ^4^ State Key Laboratory of Cellular Stress Biology, School of Life Sciences, Xiamen University, Xiamen, China; ^5^ Key Laboratory for Chemical Biology of Fujian Province, MOE Key Laboratory of Spectrochemical Analysis and Instrumentation, College of Chemistry and Chemical Engineering, Xiamen University, Xiamen, China

**Keywords:** cancer cachexia, metabolomics, metabolic alterations, progress, biomarker

## Abstract

Cancer cachexia (CC) is a complicated metabolic derangement and muscle wasting syndrome, affecting 50–80% cancer patients. So far, molecular mechanisms underlying CC remain elusive. Metabolomics techniques have been used to study metabolic shifts including changes of metabolite concentrations and disturbed metabolic pathways in the progression of CC, and expand further fundamental understanding of muscle loss. In this article, we aim to review the research progress and applications of metabolomics on CC in the past decade, and provide a theoretical basis for the study of prediction, early diagnosis, and therapy of CC.

## Introduction

Cancer cachexia (CC) is a multifactorial syndrome, which is characterized by disturbed metabolism, declined body weight, depleted muscle mass, and reduced food intake (Evans et al., 2008; Fearon et al., 2011). Overall, CC affects approximately 50–80% of cancer patients and leads to around 30% of mortality, with the highest incidence reported in gastrointestinal and pancreatic cancers (Loberg et al., 2007; Kumar et al., 2010). Lately, four stages of CC have been proposed to define the guidelines (Bozzetti and Mariani, 2009; Blum et al., 2010). Initially, CC begins in a pre-cachexia stage with unwitting body weight loss, along with a more severe and noninvertible fat tissues and skeletal muscles loss, followed by disturbances in metabolic pathway and immune system, ultimately resulting in death (Hamerman, 2002; Deans et al., 2009).

Declined body weight primarily arise from skeletal muscle loss, which is recognized as the major feature of CC. Muscle loss makes routine activities difficult and results in tiredness, in addition to the tremendous damage to quality of life and poor response to surgery or chemotherapy (Lok, 2015). Study showed that treatment on skeletal muscle loss could not only attenuate the symptoms of CC, but also remarkably prolongs lifespan (Zhou et al., 2010). Previous studies have found that CC is linked to various factors including fasting hormones, pro-inflammatory cytokines, such as interleukin 1 (IL-1), tumor necrosis factor-alpha (TNF-α), interferon-gamma (IFN-γ) (Nagaya et al., 2006; Gupta et al., 2011; Argiles et al., 2013; Argiles and Stemmler, 2013). The two main cell proteolysis pathways including ubiquitin-proteasome pathway and autophagy pathway regulate protein turnover in muscle tissues (Bonaldo and Sandri, 2013; Halle et al., 2020; Lim et al., 2020; Yang et al., 2020). In addition, several major signaling pathways including IGF1-Akt-FoxO pathway, TGFβ-myostatin pathway, NF-κB signaling, and glucocorticoids pathway have all been implicated in muscle atrophy of CC (Bodine et al., 2001; Musaro et al., 2001; Lee, 2004; Sandri et al., 2004; Waddell et al., 2008; Peterson et al., 2011). Identification of signaling pathways associated with CC and muscle atrophy has achieved great progress in recent decades. Given that CC is a typical metabolic syndrome, metabolomic techniques can be applied to explore biomarkers for early diagnosis of CC, address metabolic characteristics for mechanistic understanding of the pathogenesis of CC, and develop therapeutics strategies for treatments of CC.

As an omics technology developing after genomics, transcriptomics and proteomics, metabolomics has rapid developments at present, which can simultaneously analyze all of metabolites with small molecular weights in a biological system (Newgard, 2017). Compared to genomics, transcriptomics and proteomics, metabolomics is based on extensively used detection equipment including either mass spectrometry (MS) or nuclear magnetic resonance spectroscopy (NMR), which has the features of high sensitivity, high precision, good resolution, and small sample volume (Beckonert et al., 2007; Ma et al., 2018). In the last 2 decades, metabolomic techniques have been extensively used to exploring various diseases such as cancer (Wishart, 2016; Schmidt et al., 2021), type 2 diabetes (Newgard et al., 2009; Ma et al., 2018), fatty liver (Gao et al., 2009; Lallukka and Yki-Jarvinen, 2016), and cardiovascular diseases (Shah et al., 2012a; Shah et al., 2012b).

Metabolomic techniques create ideas and clues for scholars to predict and screen CC at early stage. Recently, researchers have applied metabolomic analysis to perform global and in-depth studies for identifying metabolic signatures in patients or animal models or cell models with CC, and also for identifying potential biomarkers and crucial metabolic pathways to mechanistically understand the pathogenesis of CC. We searched for articles from PubMed, Scopus and Google Scholar relevant to cancer cachexia by using the keywords “cancer cachexia and metabolomics,” “cancer cachexia and metabonomics,” “cancer cachexia and metabolic,” “muscle atrophy and metabolomics,” “muscle loss and metabolomics” and so on. We have only included the studies based on the animal models or clinical samples related to CC by using metabolomics methodologies. So far, only two reviews of omics studies on CC have been reported (Gallagher et al., 2016; Twelkmeyer et al., 2017). However, these reviews did not pay much attention to the field of metabolomics. To widely expand the knowledge of CC and give inspirations for the cachexia studies from the view of biomarkers, signatures and therapeutic targets, we focus on the progress made in the past decade, novel developments, and latest discoveries in the study of CC using metabolomic techniques, and look forward to its future developments. To the best of our knowledge, this article presents the first review on the progress of metabolomic applications in CC.

## Metabolomic Research Methodologies and Techniques

**FIGURE 1:**
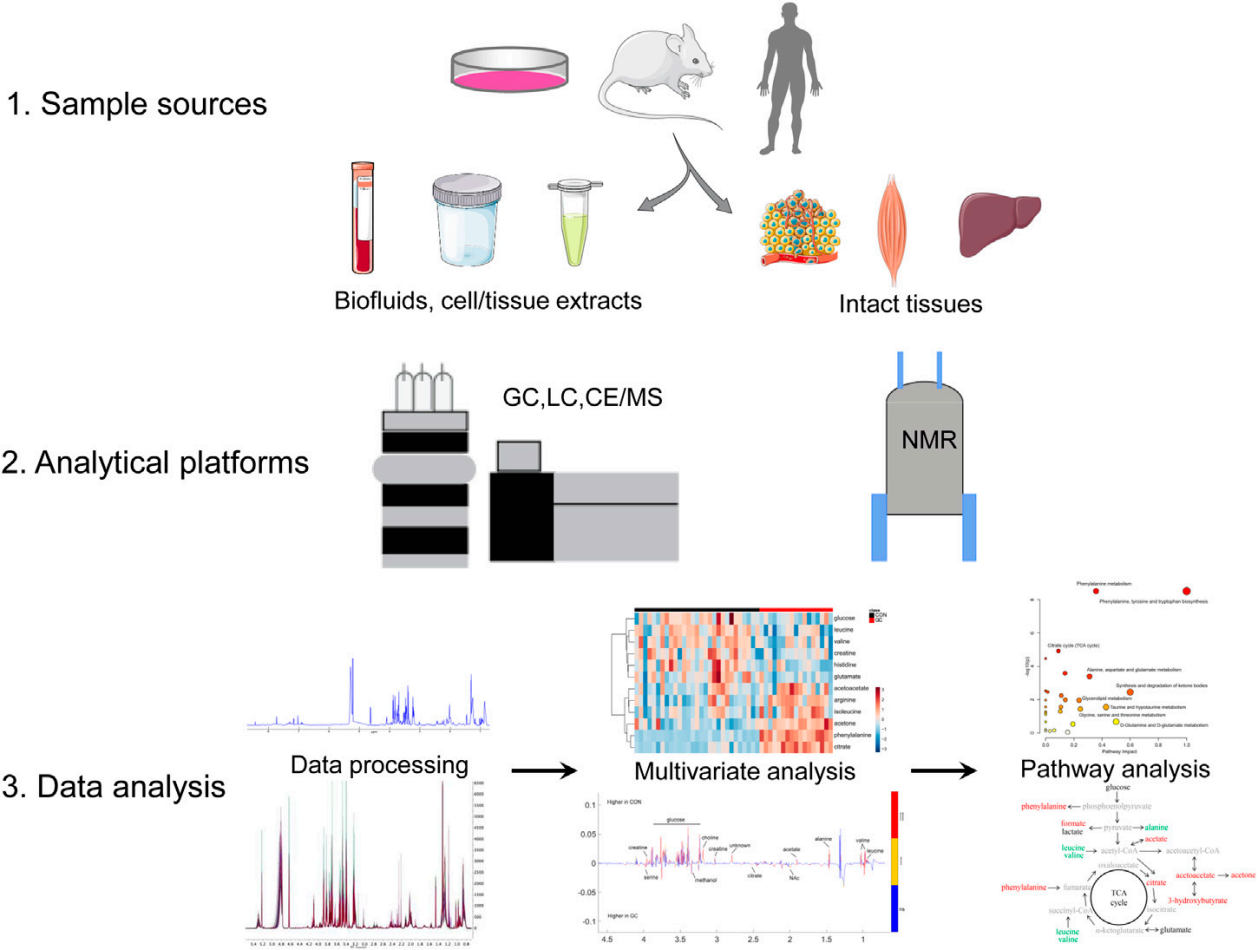
Metabolomics analysis workflow. Abbreviations: NMR, nuclear magnetic resonance; CE, capillary electrophoresis; GC, gas chromatography; LC, liquid chromatography; MS, mass spectrometry.

Followed by genomics, transcriptomics, and proteomics, metabolomics is a promising subject that has promptly developed in recent years. It can be qualitatively and quantitatively employed to analyze various sample sources, which include cells or tissues extract, bio-fluids, and microorganisms caused by genetically engineered or drug treatment. Metabolomic analyses usually focus on small molecular metabolites such as amino acids, lipids, small molecular peptides, and organic acids with a relative molecular weight of less than 1,000 Da (Nicholson et al., 1999; Fiehn et al., 2000; Xia and Wan, 2021). Generally, metabolomics includes two tools: non-targeted and targeted metabolomics. Non-targeted metabolomics is most widely used in CC studies to explore biomarkers (Yang et al., 2018), signatures (Cui et al., 2019b), and therapeutic targets (Fukawa et al., 2016). The process of metabolomic analysis in CC is depicted in Figure 1, which contains sample sources, analytical platforms, data collection and analysis, biomarkers identification, metabolic pathways exploration, and biological significance elucidation.

### Sample Sources

The most commonly used type of samples for metabolomic studies in CC are serum/plasma, urine, tumor tissues, liver and skeletal muscle, and other tissues. The collected blood samples are further processed with cell separation to obtain sera and plasma at 4°C before analysis. This step might be one of the major factors of pre-analysis errors in blood metabolomics research (Beckonert et al., 2007; Nikolic et al., 2014). It is generally suggested that the interval between blood sample collection and cell separation should be finished in 35 min to avoid the increased lactate levels. In addition, repeated freezing and thawing steps should be avoided in the whole experiment (Yin et al., 2015). Compared with blood samples, the biological composition of urine samples is relatively simple, the protein content is low, and additional metabolite extraction steps are not usually required. The commonly used pretreatment method for skeletal muscle and tumor tissues in CC studies is liquid-liquid extraction. In general, tissue samples are initially extracted with cold solutions which contains chloroform, methanol and water in a certain ratio to generate a two-phase system. The polar and non-polar metabolites are separated, lyophilized and dissolved in corresponding solvents, respectively (Jonsson et al., 2005; Beckonert et al., 2007; Legido-Quigley et al., 2010).

### Data Collection Techniques

Metabolomic techniques are applied to measure the number, type, condition, and level of metabolites and to explore metabolic profiles (Beckonert et al., 2007). Compared to other detection techniques, NMR and MS are the two mostly used techniques for metabolomic analysis (Emwas, 2015).

NMR technology is a spectroscopic technology that uses different atomic nuclei to absorb ratio-frequency radiation with different resonance frequencies, which are converted into molecular chemistry and structural information related to environments of the nuclei (Bothwell and Griffin, 2011). With the development of NMR technology, researchers can directly analyze intact gastrocnemius muscle without any pretreatment of samples by using high-resolution magic angle rotation (HRMAS-NMR) spectroscopy in a CC mouse model (Yang et al., 2015). Overall, NMR spectroscopy has many advantages such as simple sample preparation, non-invasive and unbiased measurement of the sample, good objectivity and reproducibility (Li et al., 2015). However, signal overlap and low sensitivity are two obvious shortcomings in complicated ^1^H-NMR spectra.

MS spectroscopy uses electric and magnetic fields to separate moving ions and detect them according to the mass-to-charge ratio (m/z) (Tsiropoulou et al., 2017). At present, MS combined with chromatography are divided into three types including capillary electrophoresis-mass (CE-MS), gas chromatography-mass (GC-MS), and liquid chromatography-mass (LC-MS). CE-MS has high performance for polar and ionic compounds with high resolution and sensitivity rather than uncharged compounds (Ramautar et al., 2019; Stolz et al., 2019). Compared to GC and LC, CE has a superiority over them for the resolution of charged molecules along with the isomers due to the excellent separation. GC-MS is generally conducted to analyze non-polar, low-boiling and volatile molecules, and samples usually need to be derivatized. LC-MS has relatively high sensitivity and strong detection ability for polar and thermally unstable compounds, by which a wider range of metabolites with low detection limit can be analyzed. It can be used for trace analysis and is more suitable for metabolomic analysis of complex biological samples (Römisch-Margl et al., 2011; Xiao et al., 2012). Cala and colleagues performed a combination of 3 types of MS (GC-MS, CE-MS, and LC-MS) to obtain plasma metabolite fingerprinting in a CC clinical study (Cala et al., 2018).

Compared with NMR spectroscopy, MS has several advantages such as high sensitivity and resolution, which could detect thousands of metabolites in a large dynamic range at the same time. However, MS also has its own shortcomings such as complicated sample preparation and low reproducibility. The advantages and drawbacks of MS and NMR detections are listed in Table 1. To promote the entire performance of metabolomics studies, Pin and colleagues combined MS and NMR to investigate differences between CC and chemotherapy induced cachexia (Pin et al., 2019).

**TABLE 1:** Summarization of advantages and drawbacks of MS and NMR detections.

Features	NMR	MS
Sample preparation	Simple	Complex
Sample measurement	Simple	Complex, various chromatography methods
Sample recovery	Good, non-invasive	Destructive
Selectivity and targeted analysis capabilities	General, mostly in untargeted analysis	Good, untargeted and targeted analysis
Sensibility	Low, <100 metabolites per test	High, >1,000 metabolites per test
Resolution	General	General
Repeatability	High	low

### Data Preprocessing and Analysis

Data analysis includes data preprocessing, multivariate statistical analysis, model establishment and verification, and selection of difference variables, etc. Prior to obtaining metabolomics data for statistical analysis, it is necessary to preprocess the data, which mainly includes baseline correction, peak screening (peak identification, peak alignment and correction), noise filtering, missing value processing, normalization and scaling (Dunn et al., 2011). Thereafter, multivariate statistical analysis is conducted to decrease the dimensionality of acquired data and extract information, including principal component analysis (PCA), clustering analysis, partial least square analysis (PLS), PLS-discriminant analysis (PLS-DA), orthogonal PLS (OPLS)-DA and random forests (RF) (Idborg-Bjorkman et al., 2003; Wiklund et al., 2008; Sugimoto et al., 2012; Schwammle et al., 2015). PCA, PLS-DA, OPLS loading plot and heatmap analysis were most commonly used in CC metabolomics studies. Besides, general statistical analyses including analysis of variance (ANOVA) and Student’s t-test are also applied to quantitatively analyze the abundance of metabolites between different groups. When performing the multiple comparisons, the familywise error rate (FWER) might cause false-positive detection, which could be diminished by the procedures of false discovery rate (FDR) with Holm, Bonferroni and Benjamini-Hochberg corrections in metabolomic analysis (Sugimoto et al., 2012; Muroya et al., 2020). Overall, the combination of multivariate statistical analysis and classical statistical analysis can improve the reliability of the data analysis.

### Data Elucidation

After multivariate statistical analysis, one can uncover and illustrate metabolic signatures based on several databases, including significantly altered concentrations of metabolites and certain disturbed metabolic pathways corresponding to external metabolic stimuli. These databases include HMDB (http://www.hmdb.ca/), METLIN (https://metlin.scripps.edu/), SMPDB (https://smpdb.ca), MassBank (http://www.massbank.jp/), The Kyoto Encyclopedia of Genes and Genomes (KEGG; https://www.genome.jp/kegg/), and software such as MetaboAnalyst 5.0 (https://www.metaboanalyst.ca/) (Pang et al., 2021). A growing number of studies have been using MetaboAnalyst website to conduct the pathway analysis and ROC analysis in CC studies (Yang et al., 2018; Cui et al., 2019b; Sadek et al., 2021).

## Advances in the Pathogenesis of Cancer Cachexia Based on Metabolomics

**FIGURE 2:**
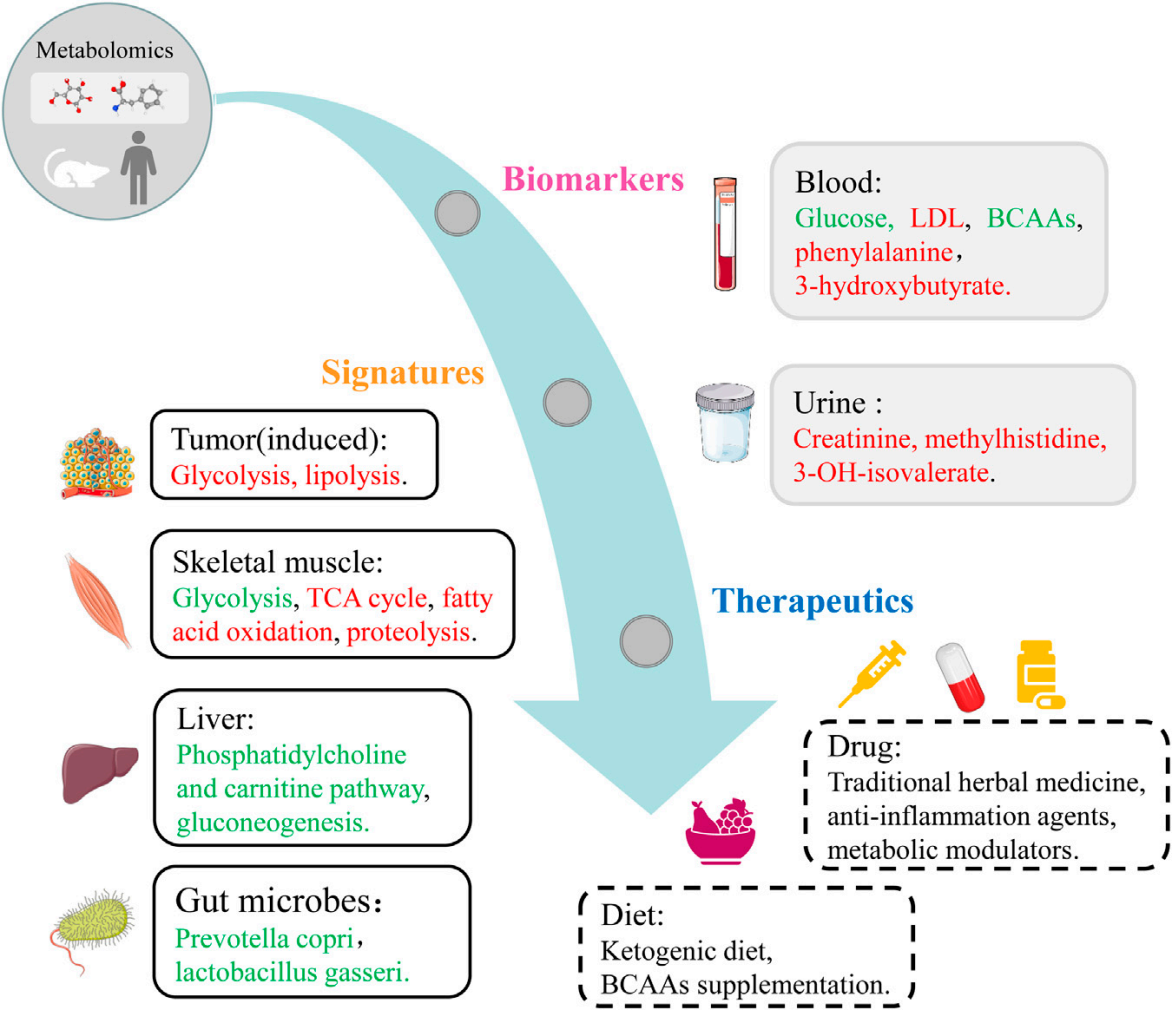
Metabolomics applications covering biomarkers, signatures and therapeutic targets in CC. Red, upregulated metabolites and pathways in CC group. Green, downregulated metabolites, microbes and pathways in CC group.

Skeletal muscle loss might occur in the early stage, which might be masked by dysfunction and symptoms of other tissues. Methods used to assess muscle loss involve diagnostic imaging techniques, including computed tomography (CT), dual energy X-ray absorptiometry (DXA), and magnetic resonance imaging (MRI). However, these methods are associated with several shortcomings such as time consuming, expensive, complicated, and invasive when clinicians wish to screen the early or slow muscle loss (Heymsfield et al., 1997; Mitsiopoulos et al., 1998; Shen et al., 2004; Mourtzakis et al., 2008). In order to exploit the progress of muscle loss dynamically, several methods have been developed to detect CC syndromes and shorten the period for early prevention (Evans et al., 2008). Recently, metabolomic analysis is widely being applied to uncover novel biomarkers, explore certain metabolic pathways associated with the pathogenesis of various diseases including CC, and ultimately exploit potential therapeutic strategies in the future (Twelkmeyer et al., 2017). Applications of metabolomics analysis in CC are depicted in Figure 2, which cover biomarkers, signatures and therapeutic targets.

### Biomarkers

Metabolomics can be applied to detect hundreds of small metabolites simultaneously for providing better elucidation of metabolic pathways related to the pathological mechanisms of CC, ultimately identifying reliable biomarkers for diagnosis and monitoring of cachexia.

Metabolomics studies in CC began in 2008 relied on the classical colon-26 (C26) mouse model. Connell and colleagues demonstrated that metabolomic analysis has the ability to diagnose and discover the surrogate serum biomarkers in CC for the first time (O'Connell et al., 2008). They conducted NMR-based metabolomic analysis on serum samples, and observed significant metabolic alterations including elevated amounts of very low-density lipoprotein (VLDL) and low-density lipoprotein (LDL) related to aberrant glycosylation of β-Dystroglycan (O'Connell et al., 2008). In a recent study based on the same C26 model, Lautaoja and colleagues identified free phenylalanine in sera and muscle tissues as a promising biomarker of cachectic muscle atrophy by using GC-MS-based metabolomic analysis (Lautaoja et al., 2019).

Furthermore, Kunz and colleagues performed untargeted LC-MS-based metabolomic analysis of plasma and skeletal muscle in a Lewis lung carcinoma (LLC) mouse model. They detected increased levels of asymmetric dimethylarginine, and NG-monomethyl-L-arginine in LLC group relative to normal group. In order to further explore the function of these two methylarginines in muscle turnover, the researchers treated the cultured myotubes with these two metabolites and found impaired muscle protein synthesis *in vitro* study. Surprisingly, increased levels of asymmetric dimethylarginine were also observed in muscle tissues from clinic patients. This study not only discovered two novel potential biomarkers, but also provided therapeutic ideas for CC (Kunz et al., 2020).

In addition, Yang and colleagues revealed dynamically changing metabolic profiles in sera and intact muscle of CC in the C26 mouse model from pre-cachexia to the refractory cachexia period. They identified five unique metabolic features including declined levels of serum glucose and BCAAs, increased levels of ketone bodies, neutral amino acids and 3-methylhistidine (Yang et al., 2015). Using HRMAS-NMR spectroscopy, they performed metabolic profiling of cachectic gastrocnemius muscle for the first time. To further validate the metabolic features identified from the mouse model, recently, Yang and colleagues recruited 33 pre-cachectic, 84 cachectic and 105 cancer patients with stable body weights and 74 healthy controls, according to the international definition and classification of CC (Fearon et al., 2011). They conducted NMR metabolomic analyses on sera and urine of CC patients to reveal the metabolic profile of CC, and identified 15 metabolites for discriminating different disease states (Yang et al., 2018). Based on three identified metabolites (carnosine, leucine and phenyl acetate), they established a diagnostic model for predicting the presence of cachexia with high accuracy.

In a previous study, Fujiwara and colleagues enrolled 21 advanced pancreatic cancer patients with or without cachexia, collected serum samples at different time point, and performed GC-MS-based metabolomic analysis (Fujiwara et al., 2014). They observed intraday differences in serum metabolite concentration, which were observably altered in the evening but basically identical in the daytime. Specifically, abundance of paraxanthine was significantly decreased in CC patients compared to those without cachexia all day long, which was potentially associated with cachexia. Additionally, another study performed NMR-based metabolomics analysis on 170 patients with head and neck squamous cell carcinoma cancer (HNSCC). These patients experienced radical treatments with radio-/chemo-radiotherapy (RT/CHRT) (Boguszewicz et al., 2019). Boguszewicz and colleagues indicated that serum metabolic alterations primarily related to high 3-hydroxybutyrate levels could be detected at an early stage of the treatment experienced by HNSCC patients. Thus, 3-hydroxybutyrate could be exploited as a fast and sensitive biomarker of malnutrition or cachexia.

Similarly, Miller and colleagues conducted LC-MS-based metabolomic analysis and identified potential biomarkers related to weight loss in patients with upper gastrointestinal cancer, which could be applied for the assessment of therapeutic intervention (Miller et al., 2019). Cancer patients with ≥5% weight loss displayed plasma metabolic profiles distinguished from those with <5% weight loss. Totally, six metabolites were highly discriminative of body weight loss, including lysoPC18.2 and 16:1, hexadecanoic acid, octadecanoic acid, phenylalanine.

Metabolites in urine samples have also been investigated to discover novel biomarkers for CC. Eisner and colleagues did the first attempt to use single time-point urinary metabolite profiles to diagnose muscle wasting occurring in CC humans (Eisner et al., 2011). After analyzing 93 random urine samples from cancer patients, the researchers found that some metabolites such as creatinine and methylhistidine arising from muscle proteolysis were particularly released into urine. This study provides an inspiration that it might also be convenient, cheap and safe to detect muscle wasting based on ^1^H-NMR urine metabolomic analysis. Overall, these results obtained from previous studies on biomarkers for CC mostly depend on the samples derived from animal models and human, and also on tumor type, bio-fluids and analytical platforms.

### Metabolic Signatures and Metabolic Pathways

Although numerous researches have been exploring molecular mechanisms underlying muscle wasting in CC, the effect of muscle wasting on muscle function and metabolic signatures remains unclear (Diffee et al., 2002). Metabolic impairments in the skeletal muscle are related to its physiological dysfunction. Thus, metabolic derangements might be involved in molecular mechanisms underlying protein synthesis and breakdown (Tisdale, 2003; Santarpia et al., 2011).

Yang and colleagues indicated that serum metabolic disturbances associated with promoted tricarboxylic acid (TCA) cycle and amino acid metabolism were the major features of CC in C26 mouse model. Amino acids, ketone bodies and metabolites involved in TCA cycle were recognized as potential biomarkers related to the corresponding metabolic pathways (Quanjun et al., 2013). Furthermore, Torossian and colleagues performed GC-MS-based and LC-MS-based metabolomic analyses to reveal metabolic distinctions between cachectic gastrocnemius muscles and control muscles in the C26 mouse model (Der-Torossian et al., 2013b). They showed predominant effects of CC including: enhanced oxidative stress, impaired redox homeostasis, altered metabolite concentrations in glycolysis and declined carbon flow through TCA cycle. This study found the tumor Warburg-like metabolic pattern in skeletal muscle of CC for the first time, which is considered as novel metabolic signature in CC research.

Compared to malabsorption, fasting, age-induced muscle loss, and sarcopenia, CC has its own metabolic features (Fearon, 1992; Tisdale, 2009; Evans, 2010; Rolland et al., 2011). Consistently, Torossian and colleagues performed a NMR-based metabolomic analysis of sera, and indicated that metabolic alterations including hyperlipidemia, hyperglycemia and reduced BCAAs distinguish cachexia from effects of starvation (Der-Torossian et al., 2013a). Another previous study explored metabolic differences between sarcopenia and CC in senile cancer animals. The researchers conducted NMR-based metabolomic analysis dynamically on sera derived from adult and ageing rats. The metabolic alterations mostly focused in several metabolic pathways, including amino acid biosynthesis which was upregulated in the aging group and downregulated in the tumor groups (Viana et al., 2020). Recently, they also performed NMR-based metabolomic analysis on gastrocnemius derived from weanling and young adult rats, aiming to explore metabolic alterations in cachectic hosts during the whole lifespan (Chiocchetti et al., 2021). They indicated that the most significant variations of metabolites such as glutamate, glutamine, glycine, and methylhistidine, might be associated with the early muscle catabolism and declined energy generation in cachectic muscles.

Chemotherapy is widely used to cancer patients in the clinical treatment, however, growing evidences have shown that several chemotherapeutic drugs could also lead to the occurrence of cachexia and deterioration of muscle mass. To date, only one metabolomics investigation has been done in chemotherapy-induced cachexia. Based on the C26 mouse model, Pin and colleagues revealed significant differences in amino acid catabolism, TCA cycle, and β-oxidation between CC and chemotherapy-induced cachexia by a combination of NMR-based metabolomics with targeted MS analysis (Pin et al., 2019).

Although skeletal muscles are the main tissue impaired dramatically in CC, other tissues such as liver and gut may also be affected and involved in the pathophysiology of this complex syndrome (Rohm et al., 2019). As an essential metabolic organ, liver regulates body energy metabolism and maintains its homeostasis. Dysfunction of liver metabolism are prone to cause promoted energy consumption in CC. Furthermore, gut microbial species play key roles in nutrients supplementation, cytokines and gut hormones regulation, and gut barrier function improvement. Based on these beneficial effects, scholars are exploring if these micrograms could act as novel therapeutic targets for CC (Valdes et al., 2018). Based on the C26 mouse model, Pötgens and colleagues explored the crosstalk in four different samples including caecal, portal vein, vena cava and liver by a combination of NMR-based metabolomics with gut gene sequencing and hepatic transcriptomics. Their results showed depressed glycolysis and gluconeogenesis, activated hexosamine pathway and phosphatidylcholine pathway, reduced abundances of hepatic carnitine and caecal acetate and butyrate, and decreased levels of aromatic amino acids (Potgens et al., 2021). Given that CC also induces anorexia and reduced food intake, Uzu and colleagues focused on studying metabolic signatures of brains and conducted a CE-MS-based metabolomic analysis on brain samples derived from a CC mouse model. They observed activated purine metabolism and increased xanthine oxidase activity in brains of cachexic mice relative to controls (Uzu et al., 2019).

Ni and colleagues conducted a comprehensive analysis on 31 patients with lung cancer by a combination of plasma metabolomics and gut microbiota metagenomics (Ni et al., 2021). For the first time, they explored gut microbiota functions in the clinical CC study, and observed remarkably decreased levels of BCAAs, methylhistamine, and vitamins in CC blood. They further discovered that increased levels of BCAAs and 3-oxocholic acid in non-CC blood were closely related to gut microbiota especially *Prevotella copri* and *Lactobacillus gasseri*, respectively. These results shed lights on molecular mechanisms underlying host-microbiota crosstalk in CC, and provided new strategies for preventing or treating CC through regulating gut microbiota in the future nutritional supplements.

Previously, preclinical mouse models (mainly C26 and LLC) were established by using subcutaneous implantation methods to conduct CC studies. Few murine models of CC with orthotopic implantation have been employed. Thus, our group established two orthotopic models including glioma cachexia and gastric cancer cachexia to mimic clinical characteristics of CC. In the first study, we conducted NMR-based metabolomic analysis to explore metabolic profiles in cachectic muscle based on a glioma induced cachexia murine model (Cui et al., 2019b). Our results indicated that significantly impaired pathways including energy metabolism, muscle protein breakdown and synthesis, and profoundly increased amino acids involved in TCA cycle anaplerotic. After that, we established a gastric CC murine model and performed NMR-based metabolomic analysis of gastric tissues (tumor), blood and skeletal muscle. Cachectic mice exhibited impaired glucose and nucleic acid metabolisms in tumor, hyperlipidemia and hypoglycemia in blood, and disturbed carbohydrate and amino acid metabolism in gastrocnemius (Cui et al., 2019a). Besides, we further explored the role of α-ketoglutarate in muscle protein turnover, and found α-ketoglutarate can alleviate the myotubes atrophy induced by glucose deprivation.

At present, only one study was performed using a combination of three metabolomics techniques (GC-MS, CE-MS, and LC-MS) to access a markedly different metabolic pattern in human plasma (Cala et al., 2018). Cala and colleagues collected two groups of plasma samples from 8 cachectic and 7 non-cachectic patients (Cala et al., 2018). Their results exhibited significantly decreased levels of amino acids and glycerophospholipids, and increased cortisol levels associated with cachexia. The disturbed metabolic pathways in CC included amino acid metabolism, aminoacyl-tRNA biosynthesis, fatty acid elongation, and TCA cycle. In another study, Stretch and colleagues investigated metabolic profiles of urine and plasma derived from 55 weight-losing patients by conducting NMR-based and direct injection MS-based metabolomics analyses. Their results indicated that large amounts of glycerophospholipids variations can be used to discover sarcopenia in cancer patients (Stretch et al., 2012). This study addressed one main issue that the variability of tissue mass might impact metabolic profiles, and thus could provide hints for the field of nutrition and metabolism studies. Overall, numerous researchers have investigated the metabolic signatures for CC from various aspects such as starvation, sarcopenia, chemotherapy, gut microbes, orthotopic implantation and analytical platforms, in order to give clues and inspirations to better elucidate the pathogenesis of CC and therapeutic targets discovery.

### Therapeutic Strategies by Using Metabolomics

As discussed above, metabolomics is being extensively used to uncover biomarkers, metabolic signatures and metabolic pathways of CC, and to exploit novel drug targets. In this section, we discuss studies on how metabolomics contributes to the discovery of new targets for therapy.

Gut microbiota could depress inflammation response and tumor development (Bindels et al., 2012). Some researchers have been exploring the roles of gut microbiome in CC and addressing certain metabolic signatures in the last section. Bindels and colleagues performed a further study on gut microbiota with the expectation of finding novel interventions for CC treatments. They integrated gene sequencing and metabolomics as well as molecular profiling of the host, so as to obtain a comprehensive view on the pathophysiology of CC (Bindels et al., 2016). The portal metabolome reflected significantly decreased glucose and lipoproteins levels, increased creatine and lactate levels. These data demonstrated that gut microbiota can impact intestinal homeostasis, confer benefits to the host, prolong survival and attenuate cachexia.

Increased expressions of inducible nitric oxide synthase (iNOS) have been observed in muscle tissues of cancer, AIDS, chronic heart failure, and COPD cachexia patients, suggesting that iNOS may be involved in the onset of cachexia under various conditions (Adams et al., 2003; Agusti et al., 2004; Ramamoorthy et al., 2009). Sadek and colleagues identified a signature of amino acids that were altered by iNOS activity in muscle by performing LC-MS-based and GC-MS-based metabolomic analyses based on the C26 murine model (Sadek et al., 2021). Notably, iNOS could significantly increase levels of arginine, lysine, tryptophan and methylhistidine, which could be decreased by inhibiting iNOS. Furthermore, they also found iNOS-induced significant decreases in levels of pyruvate, α-ketoglutarate and succinate, which were restored by KO iNOS. These results demonstrated that drug blockade or gene knockout of iNOS could rescue muscle loss and improve metabolic disorders in CC. This study provided the idea on how to use metabolomic techniques to identify potential targeted metabolic pathways. Initially, the researchers clarified metabolic alterations in animal models by conducting metabolomics analysis, thereafter they conducted genetic or pharmacological inhibition of iNOS on certain metabolic pathways including glycolysis, TCA cycle and fatty acid oxidation, which were all related to the energy production. Ultimately, they clearly elucidated the role of the iNOS/NO pathway in promoting energy crisis during cachexia-induced muscle wasting.

In addition, Ballarò and colleagues found that abnormal muscle mitochondrial function is correlated with excessive proteolysis, autophagy and mitophagy in the established CC model (Penna et al., 2019). They conducted NMR-based metabolomic analyses of skeletal muscle, liver and plasma. They identified significantly altered energy and protein metabolism such as decreased muscle NADH, increased glutamine, BCAAs and phenylalanine in tumor hosts. Partially, mitochondria-targeted compound SS-31 could modulate both skeletal muscle metabolome and liver metabolome, restore levels of alanine and ATP, as well as liver glycogen and glutathione. This study suggested that targeting mitochondrial function might be an efficient therapeutic approach for CC (Ballaro et al., 2021).

Researches have illustrated that intervening targeted metabolic pathways could attenuate CC symptoms and prevent muscle loss. Yang and colleagues investigated metabolic signatures of CC and the contribution of formoterol to serum metabolites in the C26 mouse model with NMR-based metabolomics approach. They identified several potential biomarkers including amino acids, ketone bodies and citrate cycle metabolites, which well reflected the effects of formoterol treatment (Quanjun et al., 2013). In a later study, this group conducted NMR-based metabolomic analysis based on the C26 mouse model. They exhibited that primary disturbed metabolic pathways in CC were biosynthesis of the BCAAs and glycine, serine, and threonine metabolism. Significantly, treatment with curcumin changed glycolysis with declined levels of lactate, alanine and glucose (Quan-Jun et al., 2015). In addition, Ohbuchi and colleagues exploited molecular mechanisms under the effects of rikkunshito (RKT) acting as a Japanese traditional herbal medicine (Kampo) for the treatment of CC. The researchers performed GC-MS-based plasma metabolomic analysis based on a rat model, and indicated that increased plasma glucarate following the RKT administration could delay body weight loss, reduce muscle wasting and ascites content (Ohbuchi et al., 2015). These studies shed lights on applications of traditional medicines for alleviating the progression of CC.

On the other hand, Tseng and colleagues performed in-depth assessments of anti-cachectic activities of a novel histone deacetylase inhibitor AR-42 in C26 and LLC mouse models (Tseng et al., 2015). The LC-MS-based metabolomic analysis displayed that impaired glycolysis, glycogen synthesis and protein turnover in cachectic muscle tissues, could be improved by AR-42 and maintain the homeostatic metabolism relative to controls. Furthermore, Fukawa and colleagues conducted LC-MS-based metabolomic analysis integrated with transcriptomic analysis in muscles in a subcutaneous kidney murine model. They found that tumor-secreted factors induced excessive fatty acid oxidation, leading to muscle tissue dysfunction and activated p38 pathway. Afterwards, they indicated that drug inhibition of fatty acid oxidation could ameliorate human myotubes atrophy *in vitro*, and further restore muscle mass and body weight of mice *in vivo* (Fukawa et al., 2016). This work provided the inspiration on how to use non-targeted metabolomic techniques to explore new therapeutic targets. Initially, the researchers elucidated metabolic alterations mainly related to excessive fatty acid oxidation in animal models by performing metabolomics analysis. Then, they conducted pharmacological inhibition of fatty acid oxidation based on *in vivo* and *in vitro* models. Ultimately, they successfully rescued body weight loss and muscle atrophy in CC mice.

Recently, our group performed integrative NMR-based metabolomic and transcriptomic analyses of gastrocnemius in two murine models of CC (CT26 and LLC), and evaluated the beneficial effects of amiloride for CC treatments (Zhou et al., 2021). We identified significantly impaired metabolic pathways including enhanced muscular proteolysis, suppressed glycolysis and ketone body oxidation in cachectic gastrocnemius. Our results indicated that amiloride can alleviate muscle loss and the progression of CC through blocking exosome release originated from cancer cells. Our study suggests that tumor-released exosome can be a potential target to attenuate muscle wasting during the progression of CC in the future.

In addition to the drug prevention, nutritional supplementation of metabolites such as BCAAs has been applied to improve impaired skeletal muscle metabolisms in diseases like AIDS and diabetes (Viana and Gomes-Marcondes, 2013). Furthermore, previous studies have demonstrated that leucine supplementation can promote nitrogen balance and restore muscle mass (Ventrucci et al., 2004; Salomao and Gomes-Marcondes, 2012). This team assessed if a leucine-rich diet could affect metabolic profiles of sera and tumor tissues in a rat model. The results exhibited down-regulated levels of tryptophan and lactate associated with a suppressed hypermetabolic state, and up-regulated levels of β-hydroxybutyrate and acetoacetate, which might indirectly contribute to the prevention of CC (Viana et al., 2016). Recently, this group conducted metabolomic analyses of sera and gastrocnemius derived from rats with leucine supplementation. The tumor-bearing rats displayed distinctly altered metabolic pathways including protein biosynthesis, glycine, serine and threonine metabolism, and ammonia recycling. Significantly, the leucine-rich diet rats showed attenuated Warburg effect and improved lipid metabolism (Miyaguti et al., 2020).

Ketone body supplementation might also contribute to regulation of glucose and lipid metabolism and prevent body weight loss (Kennedy et al., 2007). Shukla and colleagues addressed anti-cancerous and anti-cachectic properties of a ketogenic diet *in vitro*, and assessed the effects of ketone bodies on tumor mass and CC symptoms of mice by conducting NMR-based metabolomic analysis *in vivo* (Shukla et al., 2014). They observed reduced glycolytic flux and diminished glutamine uptake, decreased overall ATP content in tumor cells. These results suggest that treatment with ketone bodies could prevent cachexia phenotype. Collectively, we anticipate that exploitation of the global metabolome with metabolomics techniques can achieve more comprehensive knowledge of CC and discover effective therapeutic strategies.

## Conclusion

Even though metabolomics is relatively less used compared with other omics approaches, it is able to provide key information for further exploration of CC, including mechanistic understanding, potential biomarkers, metabolic signatures, and therapeutic strategies. With the rapid development and wide application of metabolomics analysis in the field of CC research, in-depth understandings of CC have been broadly expanded and systemized. Researchers propose novel hypotheses and develop approaches using metabolomic techniques, to exploit the features of CC and therapeutic targets for the treatments of CC. Metabolomics can be employed to identify potential biomarkers for screening early symptoms and monitoring the progression of CC, through measuring alterations in concentrations of hundreds of endogenous metabolites in bio-fluids and tissues derived from animal and human beings. In addition, numerous studies have shown that targeting specific metabolic pathways could regulate abnormal metabolisms induced by CC and ultimately alleviate syndromes of CC. Table 2 displays the summary of the metabolomics studies on CC in the past decade with novel discoveries.

**TABLE 2:** Overview of metabolic characteristics of CC.

References	Study object	Sample information	Analytical technology	Metabolic characteristics
O'Connell et al. (2008)	Mice	Serum	NMR	UP: VLDL/LDL;
				DOWN: glucose.
Eisner et al. (2011)	Patients	Urine	NMR	UP: creatine, creatinine,
				3-OH-isovalerate.
Stretch et al. (2012)	Patients	Urine,	NMR,	Glycerophospholipids and metabolites associated with amino acid metabolism.
		Plasma	MS	
Quanjun et al. (2013)	Mice	Serum	NMR	Enhanced citrate cycle and amino acid metabolism.
Der-Torossian et al. (2013a)	Mice	Serum	NMR	Hyperlipidemia, hyperglycemia;
				DOWN: BCAAs.
Der-Torossian et al. (2013b)	Mice	Muscle	LC-MS	Enhanced Warburg effect;
				Disrupted TCA cycle, promoted oxidative stress,
				impaired redox homeostasis.
Fujiwara et al. (2014)	Patients	Serum	GC-MS	Down: paraxanthine.
Ohbuchi et al. (2015)	Rat	Plasma	GC-MS	DOWN: glucarate;
Yang et al. (2015)	Mice	Serum, Muscle	NMR	UP: neutral amino acids, creatine, ketone bodies, 3-methylhistidine;
				DOWN: BCAAs, glucose.
Quan-Jun et al. (2015)	Mice	Serum	NMR	UP: phenylalanine;
				DOWN: BCAA, acetoacetate.
Tseng et al. (2015)	Mice	Muscle	LC-MS	Impaired glycolysis, glycogen synthesis; protein degradation.
Viana et al. (2016)	Rat	Serum,	NMR	UP: tryptophan, lactate, ketone bodies.
		Tumor		
Bindels et al. (2016)	Mice	Portal plasma	NMR	UP: creatine, lactate;
				DOWN: glucose, lipoproteins.
Fukawa et al. (2016)	Mice	Muscle, cell	LC-MS	Excessive fatty acid oxidation, enhanced oxidative stress.
Yang et al. (2018)	Patients	Serum, urine	NMR	UP: Carnosine, phenylacetate;
				Down: leucine.
Cala et al. (2018)	Patients	Plasma	LC-MS,	UP: cortisol;
			GC-MS,	DOWN: Glycerophospholipids,
			CE-MS	Sphingolipids.
Lautaoja et al. (2019)	Mice	Serum, Muscle	GC-MS	UP: phenylalanine.
Boguszewicz et al. (2019)	Patients	Serum	NMR	UP: 3-hydroxybutyrate.
Miller et al. (2019)	Patients	Plasma	LC-MS	UP: lysoPC 18.2, L-proline, hexadecanoic acid, octadecanoic acid, phenylalanine and lysoPC 16:1.
Pin et al. (2019)	Mice	Plasma, Muscle, Liver	NMR, MS	UP: low-density lipoprotein particles;
				DOWN: circulating glucose, liver glucose and glycogen.
Uzu et al. (2019)	Mice	Brain	CE-MS	Activated purine metabolism, Enhanced xanthine oxidase activity.
Cui et al. (2019a)	Mice	Tumor,	NMR	UP (tumor): pyruvate and lactate;
				DOWN (tumor): hypoxanthine, inosine, inosinate;
		Serum,		UP (serum): lactate and glycerol;
				DOWN (serum): glucose;
		Muscle		UP (muscle): α-ketoglutarate;
				DOWN (muscle): glucose.
Cui et al. (2019b)	Mice	Muscle	NMR	UP: glutamate, arginine, BCAAs;
				DOWN: glucose, glycerol,
				3-hydroxybutyrate.
Kunz et al. (2020)	Mice	Plasma,	LC-MS	UP: asymmetric dimethylarginine; and NG-monomethyl-L-arginine.
		Muscle		
Viana et al. (2020)	Rat	Serum	NMR	Promoted amino acid biosynthesis and metabolism.
Miyaguti et al. (2020)	Rat	Serum,	NMR	UP: tryptophan, phenylalanine, histidine, glutamine.
		Muscle		
Chiocchetti et al. (2021)	Rat	Muscle	NMR	Increased amino acid levels and disordered energetic metabolism.
Potgens et al. (2021)	Mice	Caecal, portal vein, liver,	NMR	Suppressed glycolysis and gluconeogenesis, hepatic carnitine and phosphatidylcholine pathway activity; activated hexosamine pathway.
		vena cava		
Ni et al. (2021)	Patients	Plasma, Gut	LC-MS	DOWN: methylhistamine, BCAAs, vitamins.
Ballaro et al. (2021)	Mice	Muscle, Liver, Plasma	NMR	UP: glutamine, isoleucine, leucine, valine and phenylalanine;
				DOWN: NADH and succinate.
Sadek et al. (2021)	Mice	Muscle	LC-MS	UP: arginine, lysine, tryptophan, and methylhistidine;
			GC-MS	DOWN: pyruvate, α-ketoglutarate and succinate.
Zhou et al. (2021)	Mice	Muscle	NMR	Enhanced muscular proteolysis, suppressed glycolysis and ketone body oxidation.

Current studies indicate that the metabolites of carbohydrates, lipids and amino acids are closely linked to the development and progression of CC (Figure 2). Carbohydrates related to CC primarily include glucose and lactate, TCA cycle metabolites such as citrate, succinate, and α-ketoglutarate. Lipids relevant to CC mainly include glycerophospholipids, LDL and lipid derivatives. Amino acids participating in the pathogenesis of CC mostly include BCAAs, phenylalanine and their metabolites. In addition, three kinds of ketone bodies and methylhistidine and its metabolites are also important substances involved in the pathological mechanisms of CC.

Significantly impaired metabolic pathways are associated with the pathogenesis of CC, including two main types: energy metabolism and amino acid metabolism (Figure 2). The metabolism of amino acids is usually disordered in CC mainly due to muscle turnover imbalance, such as BCAAs metabolism, arginine metabolism, glutamate and glutamine metabolism, phenylalanine and tyrosine metabolism. Glycolysis, fatty acid oxidation, and TCA cycle are mostly disturbed because of the shifted energy needs. Additionally, impaired metabolisms of carbohydrates and lipids contribute to the progression of CC via a series of metabolic pathways.

## Future Perspectives

Although the metabolic signatures of cachectic muscle are being investigated in the past decade, we still need to know how to elucidate the molecular mechanisms based on the results obtained from metabolomic analyses. The chemical complexity and large number of metabolites might be one of the challenges associated with metabolomic analyses. For example, metabolite compositions of sera, plasma and urine are manifestations of tumor, liver, muscle, and functions of gut microbes, type of diets, clinical cancer treatment, and other tumor-derived factors like exosomes and cytokines. Compared with other omics approaches, metabolomics has many advantages and also some drawbacks. No techniques are really flawless as a fact. Expectedly, metabolomic analyses should be integrated with other omics approaches, bioinformatics, biophysical techniques and signaling pathway analysis, which would provide comprehensive views on the complicated pathogenesis of CC, and expand our knowledge of fundamental mechanisms underlying metabolic disorder and muscle wasting. As unified workflows, inexpensive equipment, and humanized acquisition software and high throughput measurements as well as powerful computational analysis become more broadly available, metabolomics will play increasingly vital roles in the studies of molecular biosciences.
